# The role of *Aggregatibacter actinomycetemcomitans* and its leukotoxin in periodontitis

**DOI:** 10.1080/20002297.2017.1325203

**Published:** 2017-06-01

**Authors:** Anders Johansson, Rolf Claesson, Mark Lindholm, Carola Höglund Åberg, Jan Oscarsson

**Affiliations:** ^a^ Division of Molecular Periodontology, Department of Odontology, Faculty of Medicine, Umeå University, Umeå, Sweden; ^b^ Division of Oral Microbiology, Department of Odontology, Faculty of Medicine, Umeå University, Umeå, Sweden

## Abstract

*Aggregatibacter actinomycetemcomitans* is a Gram-negative periodontitis-associated bacterium that expresses a toxin that selectively affects leukocytes. Leukotoxin is encoded by an operon belonging to the core genome of this bacterial species. Variations in the expression of the leukotoxin have been reported, and a specific clonal type (JP2) of this bacterium with enhanced leukotoxin expression has been identified. Experimental studies have shown that leukotoxin, besides to kill leukocytes, also activates a substantial pro-inflammatory response in these cells. Characterization of clinical isolates from individuals with different geographic origin, age and periodontal status linked carriership of highly leukotoxic *A. actinomycetemcomitans* to disease. These highly virulent bacteria are of serotype b and have several common phenotypic and genetic features. Based on a great genetic diversity, *A. actinomycetemcomitans* can be distributed into a high number of different genotypes with various virulence capacities. It can be concluded that carriership of highly leukotoxic bacteria is associated with a significantly increased risk for initiation of aggressive forms of periodontitis.

